# Unexpected Hepatotoxicity of Rifampin and Saquinavir/Ritonavir in Healthy Male Volunteers

**DOI:** 10.1111/j.1753-5174.2009.00017.x

**Published:** 2009-03

**Authors:** Christophe Schmitt, Myriam Riek, Katie Winters, Malte Schutz, Susan Grange

**Affiliations:** * F. Hoffmann-La Roche, LtdBasel, Switzerland; † F. Hoffmann-La Roche, LtdWelwyn, UK; ‡ F. Hoffmann-La Roche, LtdNutley, New Jersey, USA

**Keywords:** Saquinavir, Ritonavir, Rifampin, Hepatotoxicity, Drug Interaction, Cytochrome P450

## Abstract

**Objectives:**

Rifampin is a potent inducer of the cytochrome P450 3A4 isoenzyme (CYP3A4) that metabolizes most protease inhibitor (PI) antiretrovirals. This study was designed to evaluate the steady-state pharmacokinetics and tolerability of the coadministration of the PIs saquinavir and ritonavir (a CYP3A4 inhibitor used as a pharmacoenhancer of other PIs) and rifampin when coadministered in healthy HIV-negative volunteers.

**Methods:**

In an open-label, randomized, one sequence, two-period crossover study involving 28 healthy HIV-negative volunteers, arm 1 was randomized to receive saquinavir/ritonavir 1000/100 mg twice daily while arm 2 received rifampin 600 mg once daily for 14 days. Both arms were then to receive concomitant saquinavir/ritonavir and rifampin for 2 additional weeks. Vital signs, electrocardiography, laboratory analyses, and blood levels of total saquinavir, ritonavir, rifampin, and desacetyl-rifampin, the primary metabolite of rifampin, were measured.

**Results:**

In arm 1, 10/14 (71%) and, in arm 2, 11/14 (79%) participants completed the first study phase; eight participants in arm 1 and nine in arm 2 went on to receive both saquinavir/ritonavir and rifampin. Following substantial elevations (≥ grade 2) in hepatic transaminases in participants receiving the coadministered agents, the study was discontinued prematurely. Two participants in arm 1 displayed moderate elevations after five and four doses of rifampin, respectively. In arm 2, all participants experienced severe elevations within 4 days of initiating saquinavir/ritonavir. Clinical symptoms (e.g., nausea, vomiting, abdominal pain, and headache) were more common and severe in arm 2. Clinical symptoms abated and transaminases normalized following drug discontinuation. Limited pharmacokinetic data suggest a possible relationship between transaminase elevation and elevated rifampin and desacetyl-rifampin concentrations.

**Conclusions:**

Although not confirmed in HIV-infected patients, the data indicate that rifampin should not be coadministered with saquinavir/ritonavir.

## Introduction

Tuberculosis (TB) remains a major cause of morbidity and death in the human immunodeficiency virus (HIV)-infected population, particularly in resource-limited areas [1]. New TB infections progress rapidly to disease in the presence of HIV infection, and HIV is the risk factor that has been most strongly correlated with the reactivation of latent TB infection [2]. Concomitant therapy of TB and HIV infection is complicated. The rifamycin antibacterials, indicated for multidrug treatment of active TB, also are potent inducers of the cytochrome P450 3A4 isoenzyme (CYP3A4), which metabolizes protease inhibitor (PI) antiretrovirals. There is a profound reduction in PI exposure (80% to 90%) [3] when rifampin, the most potent inducer of CYP3A4 among the rifamycins, is coadministered with a PI.

The PI ritonavir is a powerful inhibitor of CYP3A4. Its inhibitory effect on CYP3A4 has led to its widespread adoption, at subtherapeutic doses, as a pharmacoenhancer of other PIs. Data from a small pharmacokinetic (PK) study indicated that “boosting” doses of ritonavir could diminish rifampin's effect on CYP3A4 and increase saquinavir blood levels to the therapeutic range [4]. As a result, the US Centers for Disease Control and Prevention (CDC) guidelines [5] have suggested the concurrent administration of saquinavir/ritonavir (400/400 mg twice daily), together with nucleoside analogs, as a possible HIV treatment regimen for use with a rifampin-based TB treatment regimen.

In HIV-infected individuals not coinfected with TB, the historical saquinavir/ritonavir 400/400 mg twice-daily combination described in the CDC guidelines [5] has been superseded by saquinavir/ritonavir 1000/100 mg twice daily, a combination that yields better saquinavir exposure and fewer adverse events [6,7]. PK data also have confirmed that this smaller dose of ritonavir provides adequate concentrations of saquinavir when coadministered with rifampin [4]. Effective virological and immunological responses subsequently were reported from a small retrospective study of HIV/TB coinfected patients receiving both saquinavir/ritonavir 1000/100 mg twice daily and rifampin-based TB treatment [8]. More recently, a prospective study of once-daily HIV treatment using an investigational saquinavir/ritonavir 1600/200 mg combination also showed adequate saquinavir PK and consistent HIV treatment responses in a group of 32 patients receiving rifampin [9]. In the context of regulatory approval and the subsequent development of a more convenient 500 mg film-coated formulation of saquinavir mesylate, the present prospective study was undertaken to evaluate the steady-state PK and tolerability of both saquinavir/ritonavir 1000/100 mg twice daily and rifampin when coadministered in healthy HIV-negative volunteers.

## Methods

This single-center, open-label, randomized, one-sequence, two-arm crossover study was conducted in compliance with the Declaration of Helsinki (1996) and local Independent Ethics Committee requirements (Comité Consultative de Protection des Personnes dans la Recherche Biomédicale d'Alsace No. 1, Strasbourg, France). Written informed consent was obtained from all participants before any screening procedures were performed. The study was conducted at the Institut de Pharmacologie Clinique Roche in Strasbourg.

Twenty-eight healthy, nonsmoker, HIV-negative volunteers were recruited. All participants (26 white, 1 black, 1 Indian) were males ranging in age from 24 to 63 years. Mean body weight was 80 ± 11 kg. Participants were assessed to be healthy on the basis of medical screening, which included a physical examination, a medical history, measurement of vital signs (including electrocardiography [ECG]), and clinical laboratory tests (e.g., hematology, blood chemistry, urinalysis, and drug screening). Intake of inhibitors and inducers of CYP3A4 within 4 weeks and of any other medications within 2 weeks prior to the administration of the first dose of the study drugs was prohibited. Consumption of grapefruit and grapefruit juice was forbidden within 2 weeks prior to study drug administration and through the end of the study. During the study, no other medications were allowed with the exception of those to treat adverse events.

**Figure 1:**
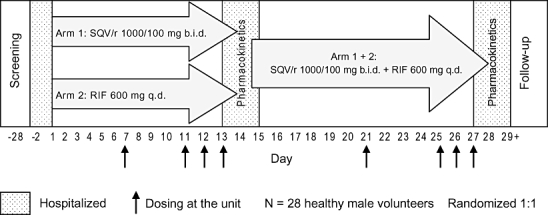
Study design.

Volunteers were randomized to receive either saquinavir/ritonavir alone (arm 1) or rifampin alone (arm 2) in phase 1 (days 1 to 14; Figure 1) of the study in order to achieve steady-state levels. Both study arms were then to receive saquinavir/ritonavir and rifampin concomitantly on days 15 through 28 (phase 2). Two saquinavir 500 mg tablets (saquinavir film-coated tablet; Invirase®; Roche) and one ritonavir 100 mg capsule (Norvir®; Abbott Laboratories) were coadministered twice daily at 12-hour intervals in the morning and evening following a standardized meal to optimize absorption. Rifampin was administered as two 300 mg capsules (Rifadin®; Sanofi Aventis) in the morning 30 minutes before breakfast and 1 hour before saquinavir/ritonavir administration. Randomization numbers were generated by Roche and provided to the study pharmacist, who assigned them sequentially in the order in which the volunteers were enrolled. Study drugs were administered under staff supervision on days when volunteers were required to return to the clinical pharmacology unit. On other days, compliance was assessed by daily phone calls and the use of diary cards.

Blood levels of saquinavir, ritonavir, rifampin, and desacetyl-rifampin—the active primary metabolite of rifampin—were to be determined on days 14 and 28. Each 5.5 mL blood sample was collected in a lithium heparin-containing tube from an indwelling catheter at:

- Predose, 0.5, 1, 2, 3, 4, 5, 6, 8, 10, 12, 14, and 24 hours after the administration of saquinavir/ritonavir for the simultaneous saquinavir/ritonavir assay
- Predose, 0.5, 1, 1.5, 2, 2.5, 3, 4, 6, 8, 10, 12, and 24 hours after rifampin administration for the rifampin/desacetyl-rifampin assay

Saquinavir and ritonavir were isolated from plasma by liquid/liquid extraction, and plasma concentrations were determined using a validated specific liquid chromatography method with tandem mass spectrometry detection. The limit of quantification was 1 ng/mL for both saquinavir and ritonavir. Plasma concentrations of rifampin and desacetyl-rifampin were determined by a validated liquid chromatography method with ultraviolet detection. The limit of quantification was 0.1 µg/mL for both rifampin and its metabolite.

Vital signs were taken and ECGs were performed at several intervals before and after drug administration. Clinical laboratory tests were performed weekly and were analyzed by a local analytical laboratory (Laboratoire Trensz, Strasbourg, France). All adverse events reported during the study were recorded and graded as to intensity.

Pharmacokinetic parameters (C_max_[peak plasma concentration], T_max_[time to C_max_], AUC_τ_[area under the concentration-time curve for the dosing interval], and 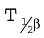
[apparent elimination half-life]) of saquinavir, ritonavir, rifampin, and its main metabolite were estimated from the concentration-time data using a noncompartmental method. No formal confirmatory hypothesis testing was planned. *P* values were to be interpreted in an exploratory manner. Safety data were evaluated descriptively. Laboratory test values were graded according to the AIDS Clinical Trial Group [10] or the American Heart Association [11] systems as appropriate or were compared with the normal range supplied by the analyzing laboratory where applicable. The upper limits of normal (ULN) were 41 IU/L for alanine aminotransferase (ALT) and 38 IU/L for aspartate aminotransferase (AST).

## Results

A total of 10/14 (71%) participants in arm 1 and 11/14 (79%) in arm 2 completed the first phase of the study (i.e., received the first 14 days of drug treatment and completed a full steady-state PK profile; Table 1). All PK parameters (Table 2) [12,13] were within expected ranges. On day 13, two participants in arm 2 experienced grade 1 elevated transaminase levels.

**Table 2:** Steady-state pharmacokinetic parameters measured/estimated on day 14 of the study

	N	C_max_(µg/mL)	T_max_(h)	AUC_τ_(µg·h/mL)	(h)
Saquinavir	10	4.54	4.00	29.1	5.55
		(44.5)	(3.00–6.00)	(54.8)	(18.3)
Ritonavir	10	1.72	4.00	10.5	4.32
		(30.6)	(2.00–5.00)	(40.2)	(29.5)
Rifampin	11	9.76	1.00	37.3	1.58
		(40.0)	(0.98–4.00)	(23.0)	(21.8)
Desacetyl-rifampin	11	0.807	2.00	4.21	2.15
		(43.7)	(1.00–4.00)	(40.2)	(30.0)

Median (min–max) for T_max_; geometric mean (CV%) for other parameters; τ = 12 hours for saquinavir/ritonavir and 24 hours for rifampin.

**Table 1:** Summary of reasons for and timing of participant withdrawal from the study

	Arm 1		Arm 2	
Reason for withdrawal	Days 1–14 Saquinavir/Ritonavir (N = 14)	Days 15–28 Saquinavir/Ritonavir + Rifampin (N = 8)	Days 1–14 Rifampin (N = 14)	Days 15–28 Rifampin + Saquinavir/Ritonavir (N = 9)
Adverse event	1	1	—	6
Consent withdrawn	1	—	1	1
Premature study termination*	4†	7	4†	2
Total number discontinued	6	8	5	9

* At the time of study termination, these patients were still receiving trial treatment.

† Two participants discontinued prior to receiving 14 days of the study drug and two participants received the full 14 days and completed a full PK profile.

**Figure 2:**
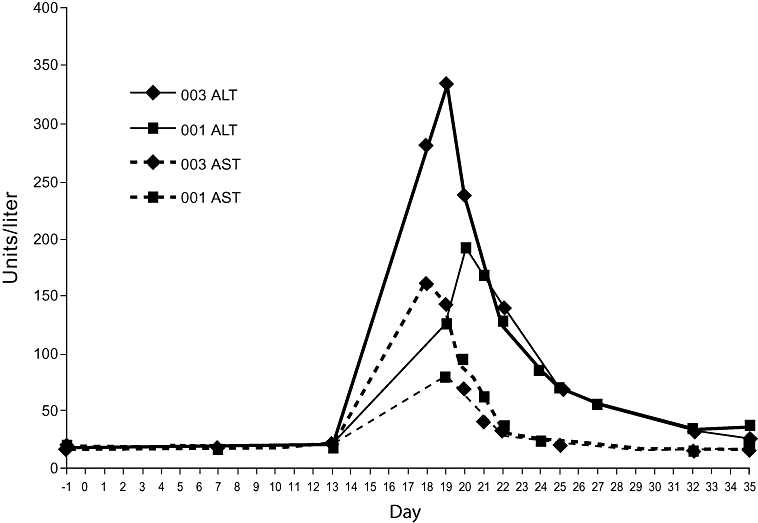
Alanine aminotransferase (ALT) and aspartate aminotransferase (AST) levels for two participants in study arm 1 who developed significant (grades 2 and 3) elevations in ALT following concomitant dosing of rifampin (600 mg once daily) with saquinavir/ritonavir (1000/100 mg twice daily). Participants took saquinavir/ritonavir alone for days 1 to 14. Falling levels are postdiscontinuation. Numbers indicated are participant identifiers.

**Figure 3:**
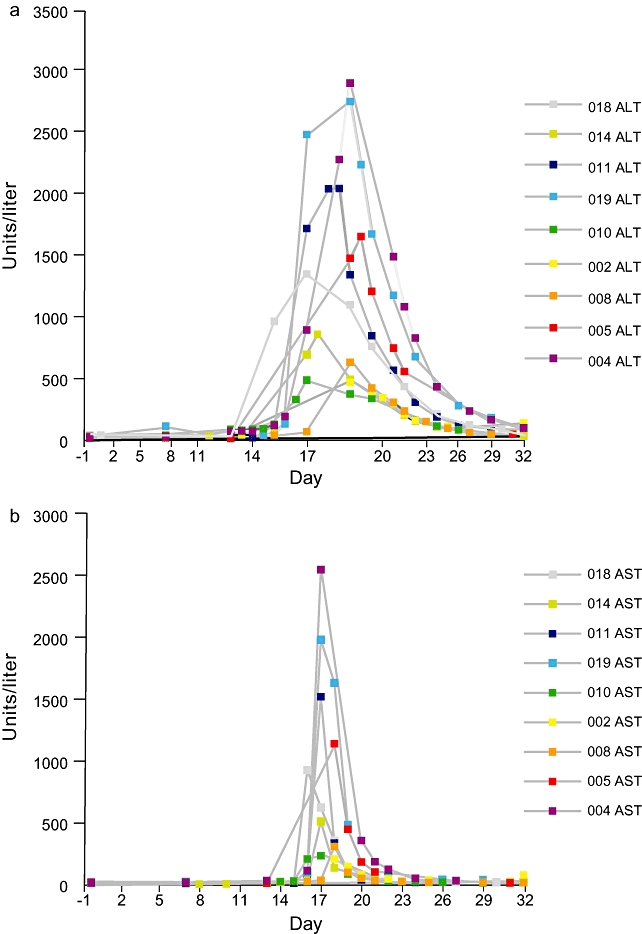
Alanine aminotransferase (ALT) and aspartate aminotransferase (AST) elevations for all nine participants in study arm 2 who commenced rifampin (600 mg once daily) + saquinavir/ritonavir (1000/100 mg twice daily) after 14 days of rifampin alone. Falling levels are post-discontinuation. Numbers indicated are participant identifiers. (a) ALT; (b) AST.

Eight participants in arm 1 and nine in arm 2 went on to receive both saquinavir/ritonavir and rifampin in the second phase of the study; however, the study was discontinued prematurely in all participants due to the occurrence of unexpected hepatic events. (Four participants from arm 1 and four from arm 2 had not received concomitant saquinavir/ritonavir and rifampin [Table 1]). Two of eight participants in arm 1 had displayed moderate elevations in ALT of 5X and 8X ULN, after five and four doses of rifampin, respectively, at the time of study discontinuation (Figure 2). The remaining six participants had received only one or two doses of rifampin. Three of these participants had normal transaminases at termination, and three showed mild ALT elevations (grade 1 or less) of 1.05 to 1.8X ULN. In arm 2, all nine participants experienced grade 4 ALT elevations of between 11X and 70X ULN within 4 days of initiating saquinavir/ritonavir (Figure 3a,b). The transaminase elevations were noted after a single dose of saquinavir/ritonavir in five of nine participants. Transaminase levels began to improve after drug discontinuation.

Clinical symptoms included nausea, vomiting, abdominal pain, and headache and were generally more common and severe for participants in arm 2 (Table 3). One participant in arm 2, who had experienced a grade 1 ALT elevation while taking rifampin alone during phase 1 of the study, was hospitalized following a 70X ULN increase in ALT after three doses of saquinavir/ritonavir. Clinical symptoms also abated following drug discontinuation. Test results for hepatitis A, B, and C, cytomegalovirus, Epstein-Barr virus, and varicella-zoster virus were all negative after study termination.

**Table 3:** Adverse events reported in >10% of participants

	Arm 1		Arm 2	
Body system/Adverse event				
Total participants with at least one adverse event	Saquinavir/Ritonavir (N = 14)	Saquinavir/Ritonavir + Rifampin (N = 8)	Rifampin (N = 14)	Rifampin + Saquinavir/Ritonavir (N = 9)
Transaminase elevation	—	2	—	9
Hepatomegaly	—	2	—	5
Chromaturia	—	—	9	—
Gastrointestinal disorder				
Nausea	4	—	1	8
Abdominal pain	2	1	1	4
Vomiting	—	—	—	4
Flatulence	1	1	—	2
Nervous system disorders				
Headache	—	—	2	—

**Figure 4:**
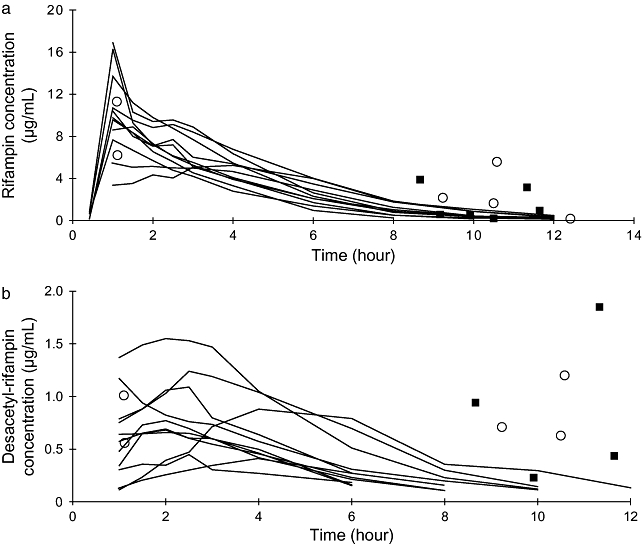
Sparse concentration–time points following initiation of the triple combination in arm 1 (○) and in arm 2 (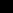) participants compared to the full pharmacokinetic profiles obtained on day 14 (arm 2; rifampin alone). (a) rifampin; (b) desacetyl-rifampin.

Although the second (day 28) PK evaluation was not performed, a few samples were available from the 17 participants who had received combined therapy during phase 2 of the study, and these were analyzed for saquinavir, ritonavir, rifampin, and desacetyl-rifampin concentrations (Figure 4). Following the coadministration of rifampin in arm 1, mean saquinavir and ritonavir concentrations were found to be similar to those observed in phase 1 of the study when these agents were administered alone (data not shown). In arm 2, the addition of saquinavir/ritonavir therapy to rifampin resulted in substantially lower saquinavir and ritonavir mean concentrations compared with the mean concentrations of saquinavir and ritonavir when administered alone in arm 1. This was consistent with both the lack of steady-state levels of saquinavir and ritonavir and the induction of CYP3A4 by two weeks of rifampin.

Data on rifampin and desacetyl-rifampin concentrations available from four participants in arm 1 were used to characterize drug disposition, and data from two additional participants were used to characterize the absorption phase only (Figure 4a, b). Of these six participants, five displayed rifampin concentrations comparable with those observed in arm 2 participants taking rifampin alone (during the first phase of the study) while the remaining participant displayed an ∼10-fold higher rifampin concentration than the mean during the first phase of the study when rifampin was administered alone (Figure 4a). This participant had not experienced elevated transaminases at the time of study discontinuation. Desacetyl-rifampin measurements were available from five participants in arm 1, two of whom showed 4-fold elevations in desacetyl-rifampin concentrations, while one showed a 7-fold elevation relative to those in arm 2 during the first phase of the study (Figure 4b).

In arm 2, a limited number of blood samples obtained from six participants during the second phase of the study provided rifampin and desacetyl-rifampin-concentration data. Four participants showed no change in rifampin levels relative to within-participant values receiving rifampin alone, although three of these four participants showed elevated desacetyl-rifampin (2- to 4-fold; Figure 4b); the remaining two participants showed elevated concentrations of both rifampin and desacetyl-rifampin. Notably in arm 2, the three participants who experienced the highest elevations in ALT (≥50X ULN) all showed elevated rifampin and/or desacetyl-rifampin. Maximum ALT increases in relation to rifampin and desacetyl-rifampin concentrations during concomitant rifampin and saquinavir/ritonavir, as determined by sparse sampling, are shown in Table 4.

**Table 4:** Elevations in alanine aminotransferase (ALT) levels in relation to rifampin and desacetyl-rifampin concentrations by participant

Arm 1/participant number	001	003	007	009	012	013	015	017	
Maximum ALT increase (X-fold the ULN)	5X	8X	1.8X	↔	1.6X	1.05X	↔	↔	
Fold increase in rifampin concentration	ND	ND	ND	↔	↔	↔	ND	10X	
Fold increase in desacetyl-rifampin concentration	ND	ND	ND	ND	4X	4X	ND	7X	
Rifampin total doses	5	4	4	1	2	2	2	1	
Saquinavir/ritonavir total doses	37	36	36	29	31	31	31	29	
Days on saquinavir/ritonavir and rifampin	5	4	4	1	2	2	2	1	
Arm 2/participant number	002	004	005	008	010	011	014	018	019
Maximum ALT increase (X-fold the ULN)	11X	70X†	40X	15X	12X†	50X	21X	33X	67X
Fold increase in rifampin concentration	ND	10X	ND	↔	↔	2X	ND	↔	↔
Fold increase in desacetyl-rifampin concentration	ND	19X	ND	2X	ND	4X	ND	ND	3X
Rifampin total doses	18	16	16	16	15	15	15	15	15
Saquinavir/ritonavir total doses	8	3	3	3	1	1	1	1	1
Days on saquinavir/ritonavir and rifampin	4	2	2	2	1	1	1	1	1

† Abnormal at end of treatment with rifampin alone.

ND = Not determined (sample taken too late with a concentration below the limit of quantification or no sample taken); ULN = Upper limit of normal; ↔ = no change.

After the study had been terminated prematurely due to these hepatic toxicities, a 6-month safety monitoring follow-up was implemented via a protocol amendment for the 17 subjects who had received triple therapy. The 6 months of additional monitoring did not reveal any evidence for persistent or new safety concerns and confirmed that the hepatotoxicity induced by the triple therapy had resolved without sequelae for all participants.

## Discussion

Coadministration of saquinavir/ritonavir 1000/100 mg twice daily and rifampin 600 mg once daily in healthy HIV-negative volunteers led to rapid and significant elevations in liver enzymes, typically within 1 to 3 days. The effect was most marked in those with steady-state rifampin concentrations at the time of saquinavir/ritonavir initiation, and in these participants the limited PK data suggested that the degree of transaminase elevation was linked to increased concentrations of rifampin and desacetyl-rifampin. The findings are consistent with a hypothesis that saquinavir/ritonavir inhibits normal metabolic breakdown of rifampin and desacetyl-rifampin and that elevated exposure exacerbates the hepatotoxic potential of rifampin. Alternatively, the administration of saquinavir/ritonavir in participants with highly induced CYP3A4 (and perhaps of other isoforms) by rifampin could have led to the rapid generation of toxic metabolite(s) of saquinavir and/or ritonavir. However, additional data are needed to confirm or refute these possibilities. It should be noted that the mechanisms underlying the hepatotoxicity of rifampicin with saquinavir/ritonavir are unlikely to be specific to the use of saquinavir; a recent trial involving rifampicin with lopinavir/ritonavir also had to be suspended because of elevated hepatic transaminases [14].

On the basis of these findings, saquinavir/ritonavir and rifampin should be considered contraindicated. A “Dear Healthcare Professional” letter to this effect was issued by Roche, the manufacturer of saquinavir, in February 2005, and a saquinavir label change acknowledged this conclusion. Still, while the findings in healthy volunteers are dramatic, hepatotoxicity of this extent and magnitude has not been reported in clinical settings where saquinavir/ritonavir and rifampin are used together in HIV-positive populations [9,15]. This drug combination has not been uncommon since the publication of the CDC guidelines for the treatment of TB in the setting of TB in 2000, and the findings reported here are puzzlingly discordant with reported data. In the prospective TBQD study [9,15], rifampin therapy was initiated 2 months before the start of saquinavir/ritonavir 1600/200 mg once daily in 30 antiretroviral-naive patients with HIV and TB and was continued for 7 months. Only two patients withdrew from saquinavir/ritonavir therapy because of elevated transaminases, and these elevations were only 5-fold above the ULN, substantially fewer patients and lower elevations than were seen in the current study in HIV-negative men who initiated saquinavir/ritonavir after achieving steady-state rifampin concentrations. Both patients with elevated transaminases had concomitant chronic hepatitis C virus (HCV) infection. Similarly, in a retrospective study of saquinavir/ritonavir 1000/100 mg in previously antiretroviral-naive patients on rifampin-containing TB treatment, only one patient out of 14 experienced a grade 3 transaminase elevation over 24 weeks and this patient was also coinfected with HCV [8].

There is additional evidence that certain drug toxicities can present differently in HIV-positive vs. HIV-negative persons [16–22]. Rifamycin-associated adverse events appear to occur less frequently in the context of HIV infection [23–26]. In particular, a greater frequency of rifabutin-associated neutropenia has been reported in HIV seronegative individuals [23–25]. Of specific relevance, the severe hepatotoxicity associated with the use of a 2-month course of rifampin and pyrazinamide to treat latent TB infection (LTBI) is uncommon in HIV-positive patients and no higher in frequency than that seen for longer term treatment with isoniazid [27–29]. By contrast, studies in largely HIV-negative populations have found a substantial increase in elevated transaminases for pyrazinamide therapy relative to isoniazid [30,31]. These rates are typically higher than those observed in studies of pyrazinamide in HIV-positive populations [29,32].

In conclusion, it is unclear why some adverse effects of drug therapy, particularly hepatic effects, appear at a higher rate in participants without HIV infection. The available evidence suggests that concomitant rifampin and saquinavir/ritonavir may fall into this category of different hepatotoxicity mechanisms between populations. Given the importance of treating TB in HIV infection and the disparity in the toxicity of combining rifampin with saquinavir/ritonavir noted between this study and the limited clinical data available, further research may be warranted.
